# Fault Diagnosis for Rolling Bearings under Variable Conditions Based on Visual Cognition

**DOI:** 10.3390/ma10060582

**Published:** 2017-05-25

**Authors:** Yujie Cheng, Bo Zhou, Chen Lu, Chao Yang

**Affiliations:** 1 School of Aeronautic Science and Engineering, Beihang University, Xueyuan Road No. 37, Haidian District, Beijing 100191, China; chengyujie@buaa.edu.cn (Y.C.); yangchao@buaa.edu.cn (C.Y.); 2 Science & Technology on Reliability and Environmental Engineering Laboratory, Xueyuan Road No. 37, Haidian District, Beijing 100191, China; 3 China Ship Development and Design Center, Zhang Zhidong Road No. 268, Wuchang District, Wuhan 430064, China; zhoubo@buaa.edu.cn; 4 School of Reliability and Systems Engineering, Beihang University, Xueyuan Road No. 37, Haidian District, Beijing 100191, China

**Keywords:** rolling bearing, fault diagnosis, variable conditions, visual cognition, speed up robust feature, isometric mapping

## Abstract

Fault diagnosis for rolling bearings has attracted increasing attention in recent years. However, few studies have focused on fault diagnosis for rolling bearings under variable conditions. This paper introduces a fault diagnosis method for rolling bearings under variable conditions based on visual cognition. The proposed method includes the following steps. First, the vibration signal data are transformed into a recurrence plot (RP), which is a two-dimensional image. Then, inspired by the visual invariance characteristic of the human visual system (HVS), we utilize speed up robust feature to extract fault features from the two-dimensional RP and generate a 64-dimensional feature vector, which is invariant to image translation, rotation, scaling variation, etc. Third, based on the manifold perception characteristic of HVS, isometric mapping, a manifold learning method that can reflect the intrinsic manifold embedded in the high-dimensional space, is employed to obtain a low-dimensional feature vector. Finally, a classical classification method, support vector machine, is utilized to realize fault diagnosis. Verification data were collected from Case Western Reserve University Bearing Data Center, and the experimental result indicates that the proposed fault diagnosis method based on visual cognition is highly effective for rolling bearings under variable conditions, thus providing a promising approach from the cognitive computing field.

## 1. Introduction

Fault diagnosis is one of the hot topics in many fields, including machinery, power chemical engineering, aviation and space. It helps reduce the loss that component and system malfunction may cause [1]. Failures of rolling bearings, which are the most widely used bearings in industry, may cause unexpected machine breakdowns and thus result in economic loss, which is an issue that has attracted considerable attention of specialists and scholars [2].

Recently, various fault diagnosis have been proposed, including expert systems, spectrum analysis, fuzzy logic, statistical processing and so on [3,4]. Among these different processing techniques, vibration-based measurement is the most widely employed due to its high correlation with structure dynamics [5,6], and the vibration signals contain a wealth of information of the bearings. However, the working environment of rolling bearings is generally tough, complex, and especially variable, which always makes the fault diagnosis methods less effective. Currently, rolling bearing fault diagnosis is the subject of intensive research, and many methods of fault diagnosis have been proposed [7], but most of these methods are based on the assumption that the rolling bearings are under fixed conditions in the execution of fault diagnosis and that the application of these methods in variable conditions is beyond their capability. Among the mass of research on rolling bearing fault diagnosis, only a few papers have considered variable conditions. Tian et al. [7] proposed rolling bearing fault diagnosis under variable conditions and utilized local mean decomposition (LMD)-singular value decomposition (SVD) to extract features; however, LMD also has the problem of iterative calculation capacity, frequency aliasing, the end effect and other issues. Mishra et al. [8] utilized angle synchronous averaging of wavelet de-noised estimates to perform rolling bearing diagnosis under variable speed conditions; however, the disadvantage of wavelet de-noising is that this method must make full use of the prior information of the signal and noise to determine the threshold.

The research of fault diagnosis has developed over many years, and the traditional rolling bearing fault diagnosis methods based on vibration processing usually consist of three steps: (1) the collection of the rolling bearing vibration signals; (2) the extraction of the fault features; and (3) rolling bearing fault diagnosis through all types of classifiers, among which the last two are the core steps [9]. Many traditional methods of feature extraction for bearing vibration signals have been proposed, such as empirical mode decomposition (EMD), short-time Fourier transform (STFT), local mean decomposition (LMD), wavelet packet transform (WPT), spectrum analysis, and so on [10,11,12,13]. However, EMD has the drawbacks of over envelopment, less envelopment, end effect and frequency confusion [14]. Additionally, STFT is unable to simultaneously meet the requirements of resolution and time [15]. LMD also has the problem of frequency aliasing and end effect. For WPT, the prior knowledge of signals is needed, which could help us to choose the correct wavelet basis [16]. In view of the problems of the above methods, we need to research a new method of bearing vibration signal feature extraction on the basis of the non-stationary and nonlinear bearing vibration signals to achieve feature extraction under variable conditions.

Cognitive science is an interdisciplinary research covering various fields, including psychology, neuroscience, linguistics, philosophy, computer science, anthropology, sociology, and biology [17]. A great number of studies have investigated the sensory organ cognitive ability of human beings, among which visual cognition has become the hot topic in cognitive science in recent years [18]. Currently, visual cognitive computing has been applied in many fields, including face recognition [19,20], gesture recognition [21,22,23], handwritten numeral recognition [24,25,26], etc. Essentially, visual cognition for identification involves the use of bionics science, which is based on recognition of the human visual system (HVS) for identification. One of the important characteristics of visual cognition is the visual invariance characteristic (VIC), i.e., the visual recognition of objects regardless of their relative spatial orientation. Our visual system is able to recognize objects from various viewing angles, orientations, deformations, and scales and in different lighting conditions [27,28]. Inspired by VIC, in this article, the author transforms the vibration signals of different fault modes under different working conditions into two-dimensional images and extracts stable fault features based on the VIC of the HVS to realize fault diagnosis under variable conditions.

Seung et al. noted that the brain stores images as a manifold of which the dimensionality is equal to the number of photoreceptor cells in the eyes; this is another characteristic of the HVS referred to as the manifold perception characteristic (MPC) [29]. Manifold learning has emerged in nonlinear feature extraction for its capability in effectively identifying low-dimensional nonlinear structure hidden in high-dimensional data [30,31]. Therefore, this article employs manifold learning to structure a low-dimensional intrinsic manifold, which can express an essential space position of the extracted features in a better way. In this paper, features extracted by VIC are expressed in low-dimensional space through manifold learning.

This paper is structured as follows: Section 2 introduces the image transformation method, which generates images for visual cognition. Then, the VIC of the HVS and its corresponding computing method are described. The nonlinear dimensionality reduction method, isometric mapping (Isomap), which is derived from the MPC of the HVS is also introduced. Section 3 describes the entire method of fault diagnosis for rolling bearing under variable conditions, including description of the experimental data, image transformation, feature extraction and fault classification. Section 4 provides the results and discussion using the data from the Case Western Reserve University Bearing Data Center. The conclusions are stated in Section 5.

## 2. Related Theories

### 2.1. Recurrence Plot

The prior work of introducing visual cognitive theory into the field of rolling bearing fault diagnosis is to achieve the transformation of unidimensional vibration signals into two-dimensional images. Image transformation is an important basis of successfully ensuring the extraction of features using VIC. Therefore, selection of a suitable image transformation method is especially important. On account of the nonlinear and non-stationary characteristics of rolling bearing signals, detection of dynamical changes in complex systems is one of the most difficult problems. Recurrence plot (RP), i.e., developing two-dimensional graphical plots that show the recurrence of states, is an important method for detecting dynamical changes [32]. It can uncover hidden periodicities in a signal in the recurrence domain that are not easily identified, and it is an important method for analyzing the periodic, chaotic and non-stationary properties of time series. This paper translates the vibration signals under variable conditions into RPs and extracts VIC features.

#### 2.1.1. Recurrence Plot Theory

The recurrence plot algorithm utilized the concept of phase-space reconstruction, which can be described as follows:

(1) For the time series $u_{k}(k=1,2,\cdots N)$, whose sample interval is Δt, we choose the Mutual Information Method and CAO algorithm to calculate the suitable embedding dimensionality m and delay time τ, which could reconstitute the time series. The reconstructed time series is as follows:
  (1) $$x_{i}=\left(u_{i},u_{i+\tau },\cdots ,u_{i+\left(m-1\right)\tau }\right),i=1,2,\cdots N-\left(m-1\right)\tau$$
(2) Calculate the norm (e.g., the Euclidean norm) of ith $x_{i}$ and jth $x_{j}$ in reconstructed phase space [33]:
  (2) $$S_{ij}=\left\|x_{i}-x_{j}\right\|,i=1,2,\cdots N-\left(m-1\right)\tau ;j=1,2,\cdots N-\left(m-1\right)\tau$$
(3) Calculate the recurrence value [34]:
  (3) $$R\left(i,j\right)=H\left(\varepsilon_{i}-S_{ij}\right),i=1,2,\cdots ,N;j=1,2,\cdots ,N$$
  where ε is a predefined cut-off distance and H{r} is a Heaviside function:
  (4) $$H\left(r\right)=\left\{ \begin{array}{l} 1\;r\geq 0\\ 0\;r<0 \end{array}\right.$$
(4) Utilize a coordinate graph with an abscissa of i and ordinate of j to draw R(i,j), where i and j are the labels of the time series, and the image is an RP.

The details of the Mutual Information Method and CAO algorithm are as follows.

(1) Mutual Information Method

The mutual information method is an effective method to estimate the delay time, which is widely used in the phase space reconstruction.

The Shannon theory shows that we can obtain the information content of $a_{i}$ from event $b_{j}$:

(5) $$I_{AB}\left(a_{i},b_{j}\right)=\log_{2}\left[\frac{P_{AB}\left(a_{i},b_{j}\right)}{P_{A}\left(a_{i}\right)P\left(b_{j}\right)}\right]$$

The relationship between $a_{i}$ and $b_{j}$ can be expressed with the comentropy $I_{AB}$:

(6) $$I_{AB}=\sum_{ij}P_{AB}\left(a_{i},b_{j}\right)\log_{2}\left[\frac{P_{AB}\left(a_{i},b_{j}\right)}{P_{A}\left(a_{i}\right)P\left(b_{j}\right)}\right]$$

Applying the theory of mutual information, set A is

(7) $$\left\{A:a_{i}=x_{i}=x\left(t_{0}+i\tau_{x}\right)\right\}$$

and set B is

(8) $$\left\{B:b_{i}=x_{i}=x\left(t_{0}+i\tau_{x}+\tau \right)\right\}$$

Then, Equation (6) translates into

(9) $$I_{AB}\left(\tau \right)=\sum_{i}P\left[x\left(t_{0}+i\tau_{x}\right),x\left(t_{0}+i\tau_{x}+\tau \right)\right]\times \log_{2}\left\{\frac{P\left[x\left(t_{0}+i\tau_{x}\right),x\left(t_{0}+i\tau_{x}+\tau \right)\right]}{P\left[x\left(t_{0}+i\tau_{x}\right)P\left[x\left(t_{0}+i\tau_{x}+\tau \right)\right]\right]}\right\}$$

Usually at the beginning, $I_{AB}\left(\tau \right)$ is very large, so we can obtain an infinite amount of information in x(t)=x(t+0). $x\left(t_{0}+i\tau_{x}\right)$ and $x\left(t_{0}+i\tau_{x}+\tau \right)$ are completely independent for chaotic signals when τ is large; when τ→∞, I(τ)→0. We generally select the first minimum of mutual information entropy $I_{AB}\left(\tau \right)$ as the delay time.

(2) CAO algorithm

The CAO algorithm has excellent properties in that the algorithm can clearly distinguish real signal and noise and has a high computational efficiency. Firstly, we calculate the distance of the points under the embedded dimensionality:

(10) $$a\left(i,m\right)=\frac{\left\|u_{i}\left(m+1\right)-u_{n\left(i,m\right)}\left(m+1\right)\right\|}{\left\|u_{i}\left(m\right)-u_{n\left(i,m\right)}\left(m\right)\right\|},i=1,2,\cdots ,N-m$$

where ‖·‖ is the ∞ norm of the vector; $u_{i}\left(m+1\right)$ is the ith vector after phase-space rebuilding, and the embedded dimensionality is m+1; $u_{n\left(i,m\right)}\left(m+1\right)$ is the nearest vector from $u_{i}\left(m+1\right)$.

Next, calculate the average value of distance change under the same dimensionality:

(11) $$E\left(m\right)=\frac{1}{N-m}\sum_{i=1}^{N-m}a\left(i,m\right)$$

where E(m) is the average value of all a(i,m).

Last, according to the discriminant equation,

(12) $$E_{1}\left(m\right)=\frac{E\left(m+1\right)}{E\left(m\right)}$$

when $m>m_{0}$, $E_{1}\left(m\right)$ stops changing or changes slowly, and $m_{0}+1$ is the minimum embedding dimensionality.

#### 2.1.2. Comparison between Recurrence Plot and Bi-spectrum

Nowadays, many methods have been proposed to transform vibration signals to two-dimensional images, including the spectrum analysis method, among which bi-spectrum has been extensively used in fault diagnosis [35]. However, the research of bi-spectrum analysis method mostly focuses on fault diagnosis in single working condition. Fault diagnosis methods for rotating machines working under variable working conditions based on bi-spectrum analysis method have been rarely reported. Moreover, bi-spectrum method is not useful in processing non-stable signals, which limits its application. Considering the advantages of recurrence plot in dealing with non-stable and non-linear signals, the application of recurrence plot in the field of fault diagnosis has attracted more and more attention of the researchers. To investigate the influence of working condition variation to recurrence plot and bi-spectrum, the authors conduct a simulation for comparison.

**Figure 1:**
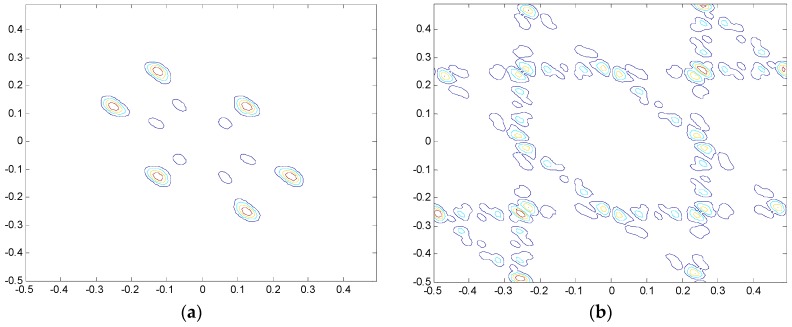
Simulation results of bi-spectrum under variable working conditions: (**a**) bi-spectrum analysis of signal $x_{1}$; and (**b**) bi-spectrum analysis of signal $x_{2}$.

**Figure 2:**
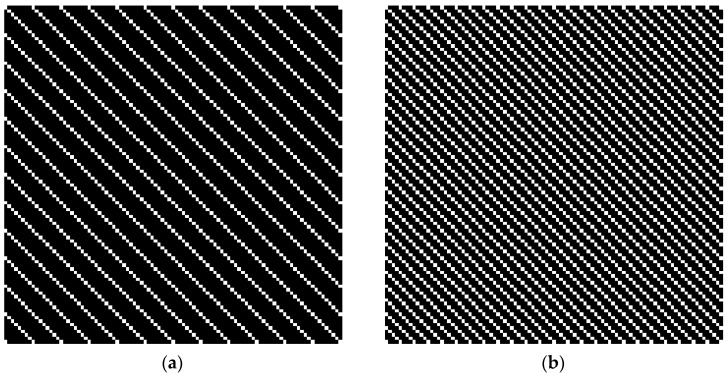
Simulation results of recurrence plot under variable working conditions: (**a**) Recurrence plot analysis of signal $x_{1}$; and (**b**) Recurrence plot analysis of signal $x_{2}$.

Given the fact that the frequency of the rolling bearing vibration signal changes when different faults occur, this study employs two sine functions of different frequency, $x_{1}=\sin\left(2\pi t/8\right)$ and $x_{2}=\sin\left(2\pi t/4\right)$, to simulate different fault types of rolling bearings. The simulation data are collected with a sampling frequency of 1 Hz and a sampling time of 100 s. Figure 1 and Figure 2 show the simulation results of bi-spectrum and recurrence plot, respectively.

In Figure 1 and Figure 2, it can be seen that, when the frequency of the sine function varies, i.e., the working condition of rolling bearing varies, the bi-spectrum figures exhibit notable variation in figure structure, whereas the recurrence plots maintain stable structure. The slight changes in two recurrence plots can be considered as scale zooming. The analysis results indicate that recurrence plot is more suitable for processing non-stationary and non-linear vibration signals.

### 2.2. VIC of the HVS and SURF

#### 2.2.1. VIC Theory

The HVS, which is the most intuitive tool to perceive the world, has recently gained considerable attention in the field of image processing [36]. Scientists believe that the essence of visual recognition is the perception of the invariant structure characteristics in the observed objects. Cayley et al. first introduced the theory of Algebraic Invariants and introduced it into the field of computing method, which initially formed the theory of visual invariance. At the Iceland conference in 1991, the theory of VIC was formally proposed [37]. The main concept of the theory is that: (1) images are composed of edge and texture details; and (2) the invariant is the essential description of the geometric structure of the object. The invariant is the most important geometric structure in the visual object, as it plays a key role in the recognition of the object.

**Figure 3:**
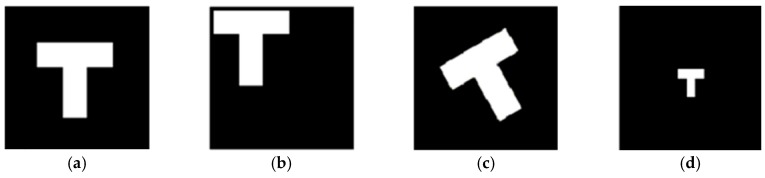
The same image shown with changes in translation, rotation and scale variation: (**a**) original image; (**b**) translation; (**c**) rotation; and (**d**) scaling.

**Figure 4:**
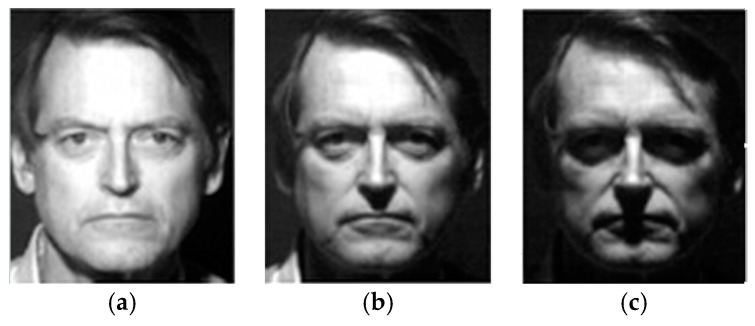
The same image shown with changes in brightness: (**a**) original image; (**b**) slight change in brightness; and (**c**) obvious change in brightness.

The main reason why the theory of visual invariance is widely employed is that it is similar to the visual essence of human beings. Human visual perception is based on invariant features, meaning that the perception and recognition of human eyes to external objects cannot change with the rotation, scale variation, translation and brightness changes in the object, as shown in Figure 3 and Figure 4. This is the most significant characteristic of the HVS [38], which indicates that human eyes recognize and understand the object based on the characteristic information of the object itself, and this does not change with rotation or scaling. It is precisely because of human vision capturing the invariant of the same target that people can recognize objects.

Because of the influence of bionics to scientific progress, humans began to study visual cognition several decades ago. Visual object classification has been a long-term interest due to its important role in a variety of applications [39]. Because rolling bearings under variable conditions of the same failure mode may display similar image characteristics, we choose an image translation method and employ the VIC of the HVS to extract the invariant features of the same failure mode under different conditions.

#### 2.2.2. SURF Theory

Recognition of the images that are rotating, scaling and translating refers to finding the stable points of the images. These points, such as corners, blobs, T-junctions and light spots in dark regions, do not disappear with rotating, scaling, translation and brightness changes. Scale-invariant feature transform (SIFT) is the corresponding computing method of VIC that can identify the invariant features and realize image matching. In 2006, Herbert Bay et al. improved SIFT and presented a novel scale and rotation invariant detector and descriptor called the speed up robust feature (SURF). SURF approximates or even outperforms previously proposed schemes with respect to repeatability, distinctiveness, and robustness, yet can be computed and compared much faster [40].

1. Theory of Scale Space

Scale space was first proposed by Iijima in 1962, and after the popularization by Witkin and Koenderink, scale space gradually gained attention and became widely used in the field of computer vision. The basic theory of scale space is that a scale parameter is introduced into the pattern information model and the scale space under multi-scale is obtained by continuously changing the scale parameter. The principal contours are extracted as eigenvectors to realize the detection of margins and corners and the extraction of different resolutions.

2. Integral Image Generation

Integral images, which can be used to fleetly conduct the function of box type convolution filtering is one of the main advantages of SURF. The entry of an integral image $I_{\sum }\left(x\right)$ at a location $x={\left(x,y\right)}^{T}$ represents the sum of all pixels in the input image I within a rectangular region formed by the origin and x [36].

(13) $$I_{\sum }\left(x\right)=\sum_{i=0}^{i\leq x}\sum_{j=0}^{j\leq y}I\left(i,j\right)$$

**Figure 5:**
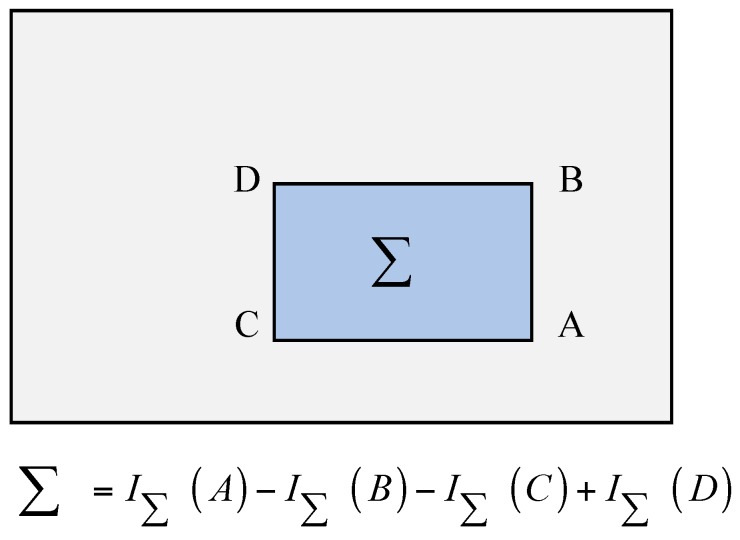
Functionality of the integral image.

Once the integral image has been computed, it takes three additions to calculate the sum of the intensities over any upright rectangular area, as shown in Figure 5 [41]. Hence, SURF uses block filtering instead of Gauss filtering, and it can greatly improve the efficiency of the algorithm.

3. Interest point localization

SURF utilizes the local maximum value of the approximate Hessian matrix of the determinant to locate the interest points. When the Hessian determinant is the local maximum, the detected point is the interest point. At the point x(x,y), which is in the original image, the Hessian matrix H(x,σ) with a scale of σ at x is defined as follows:

(14) $$H\left(x,\sigma \right)=\left[\begin{array}{l} L_{xx}\left(x,\sigma \right)\;L_{xy}\left(x,\sigma \right)\\ L_{xy}\left(x,\sigma \right)\;L_{yy}\left(x,\sigma \right) \end{array}\right]$$

where $L_{xx}\left(x,\sigma \right)$ is the convolution of the Gaussian second-order derivative $\frac{\partial^{2}}{\partial x^{2}}g\left(\sigma \right)$ with the image I at point x, and similarly for $L_{xy}\left(x,\sigma \right)$ and $L_{yy}\left(x,\sigma \right)$.

**Figure 6:**
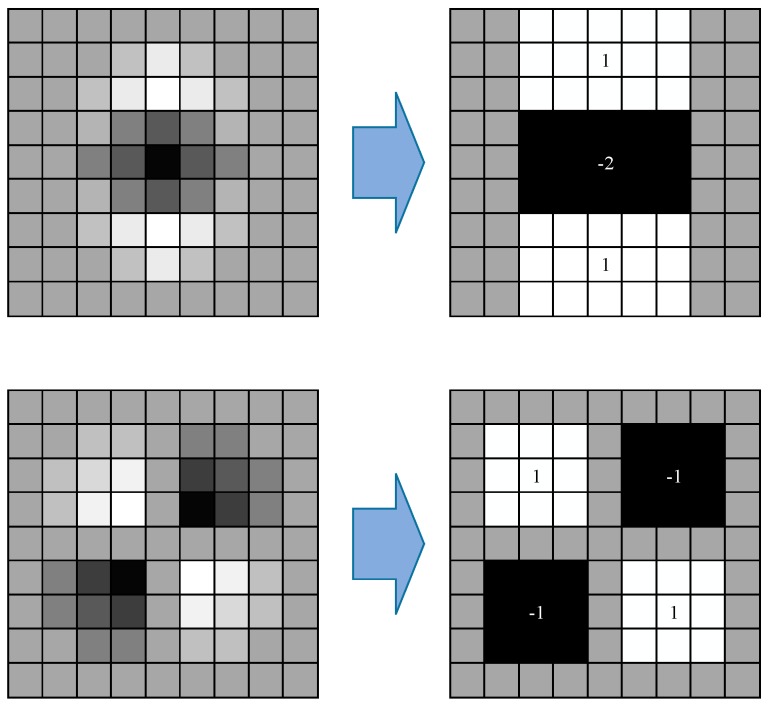
Box filter used to approximate the second-order Gaussian partial derivation.

Simple box filters using the integral image are used to approximate the second-order Gaussian partial derivation and have less computation burden, as shown in Figure 6. Box filters can be quickly calculated by the integral image, and the calculation amount is not related to the template size, which improves the computational efficiency of SURF.

When we use σ=1.2 of the second-order differential Gaussian function to filter and a template size of 9×9 as the smallest scale space to detect the points, the determinant of the Hessian matrix is

(15) $$\mathrm{Det}\left(H\right)=L_{xx}L_{yy}-L_{xy}L_{xy}$$

After simplification, the matrix becomes

(16) $$\mathrm{Det}\left(H_{approx}\right)=D_{xx}D_{yy}-{\left(0.9D_{xy}\right)}^{2}$$

where 0.9 is used to balance the Hessian determinant.

**Figure 7:**
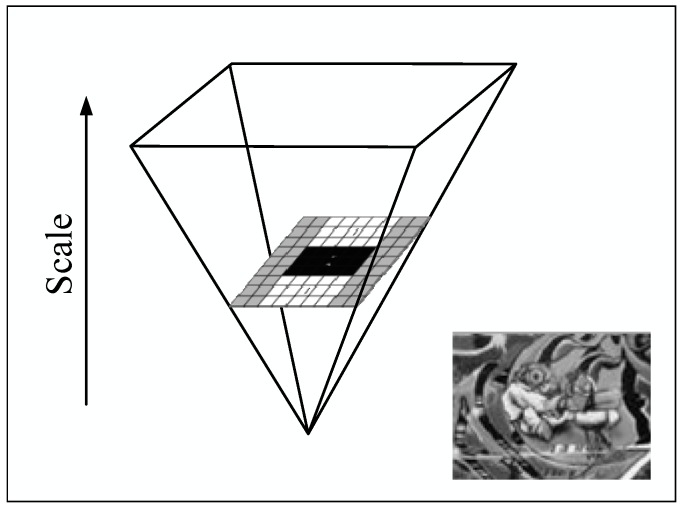
Scale pyramid.

To realize the scale invariance of the interest points, SURF applies box filters of different scales on the original image to obtain the Hessian matrix response in terms of structures of the scale pyramid, as shown in Figure 7.

As in the difference of Gaussian picture of SIFT, there are many layers referred to as octaves in the resolution pyramid, and several pictures of different scales remain in an octave. The size of pictures is unaltered, and the pictures in different octaves are obtained by changing the box filter size. In this way, SURF saves time during the down-sampling and improves the operation efficiency.

**Figure 8:**
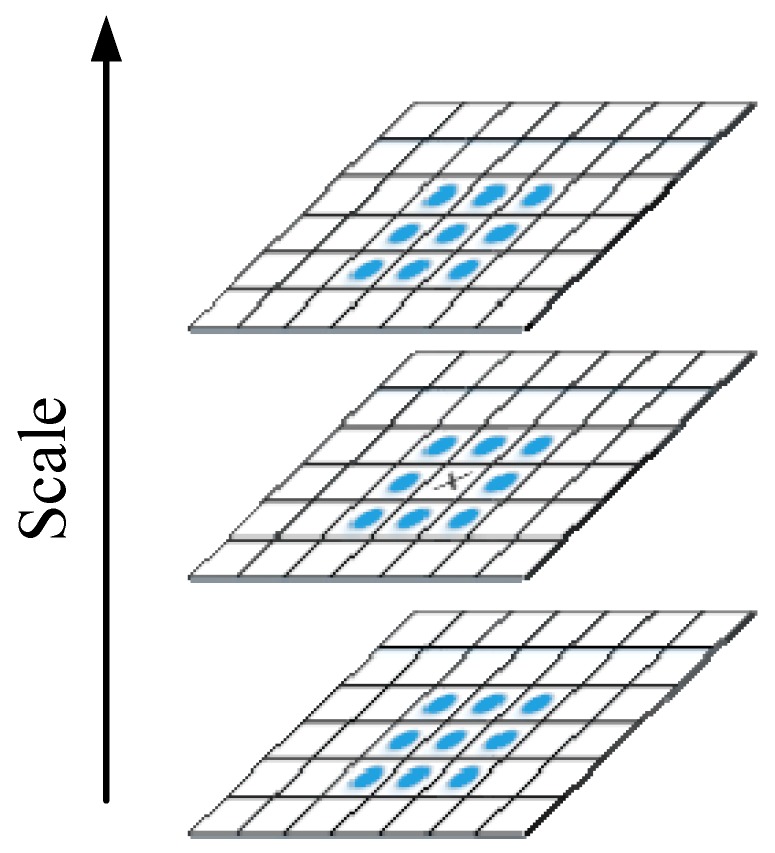
Interest point localization.

To determine the interest points, non-maximum suppression in a 3×3×3 neighborhood, which is shown in Figure 8, is employed. Each pixel processed by the Hessian matrix is compared with 26 points of its three-dimensional neighborhood to obtain the maximum or minimum as the preliminary feature points.

**Figure 9:**
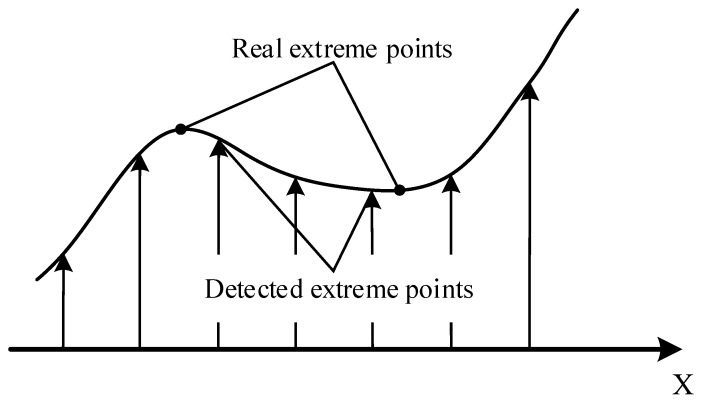
Difference between discrete space and continuous space.

The extreme point of discrete space is not a real extreme point. Figure 9 shows the difference of the extreme point of a two-dimensional function in discrete and continuous space. SURF utilizes the linear interpolation method to obtain accurate interest points.

4. Interest Point Description

**Figure 10:**
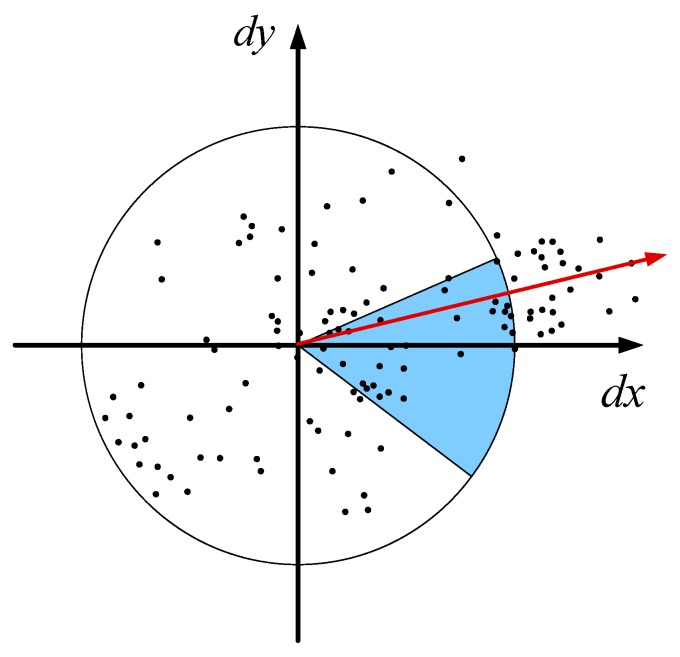
Orientation assignment of the interest point.

To guarantee the rotation invariance, the main directions of all of the interest points are required. We first calculate the Haar wavelet responses in the x and y directions within a circular neighborhood with a radius of 6σ around the interest point, where σ is the scale at which the interest point was detected [40]. Regarding the interest point as the center, the sum of all points of the Haar wavelet responses within a sliding orientation window of size 60° are the new vector. The longest such vector over all windows defines the orientation of the interest point, as shown in Figure 10.

**Figure 11:**
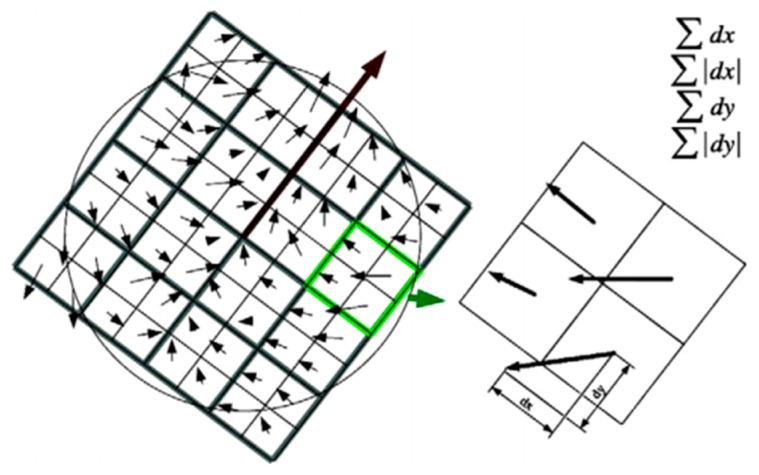
Description of the interest point.

The description of the interest point needs to split up the region regularly into smaller 4×4 square sub-regions. For each sub-region, we compute the Haar wavelet responses at 5×5 regularly spaced sample points. Finally, Haar wavelet processing is performed in each area to calculate the Haar wavelet response in the x and y directions, i.e., $d_{x}$ and $d_{y}$, as shown in Figure 11. Then, the wavelet responses $d_{x}$ and $d_{y}$ are summed over each sub-region and form the first set of entries in the feature vector, thus the four-dimensional descriptor vector $v=\left(\sum d_{x},\sum \left|d_{x}\right|,\sum d_{y},\sum \left|d_{y}\right|\right)$ is obtained. Concatenating this for all 4×4 sub-regions results in a descriptor vector with a length of 64.

### 2.3. MPC of the HVS and Isomap

#### 2.3.1. MPC and Manifold Learning

It is generally known that through the visual system, humans can receive a great wealth of information of the surrounding world with changes in visual angles and objects. In fact, neurophysiologists have found that trigger rates of nerve cell groups can be described by functions consisting of a small number of variables. They consider that the neuronal population activity is controlled by the underlying low dimensional manifold structure [42]. According to the theory of manifold perception, the visual perception process is based on manifold topological continuity. When the scale, position, illumination, and other factors are changed continuously, the image set by the same object will be located on a low-dimensional manifold in the high-dimensional observation space. Furthermore, Ref. [18] noted that the HVS receives images through visual cells, and the information received by each visual cell lies on a high-dimensional manifold space; however, the brain only receives partial information, which lies on the low-dimensional manifold. That is, the HVS has the ability to sense the information hidden in the high-dimensional manifold. Manifold learning can find meaningful low-dimensional structures hidden within high-dimensional observations, and this concept has attracted increasing research. Inspired by MPC, when the features extracted by VIC are processed, the manifold learning method can be employed to discover the essential manifold features.

Manifold learning, the most important part of the nonlinear dimensionality reduction method, has attracted widespread attention. This method can embed high-dimensional samples into low-dimensional feature space by preserving some local or global geometric structures. In recent years, many manifold learning methods have been proposed, including Isometric mapping (Isomap), Locally Linear Embedding (LLE), Laplacian Eigenmaps (LE) and Local tangent space alignment (LTSA) [43]. Isomap is the method utilized in this paper for the remaining globalized features.

#### 2.3.2. Isomap Theory

**Figure 12:**
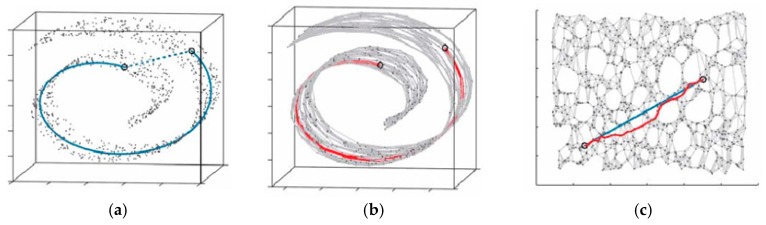
The geodesic distance in Swiss Roll. (**a**) real geodesic distance of the two samples; (**b**) approximate geodesic distance of the two samples; (**c**) comparison between the real geometric distance and the approximate geometric distance.

Isomap, which is based on the classical multidimensional scaling (MDS) method, is a global nonlinear manifold dimensionality reduction method. MDS utilizes Euclidean distance as the measure of data points, however Isomap strives to retain the intrinsic geometric characteristics of the data by acquiring the geodesic distance between all points. This method preserves geodesic proximities using a non-Euclidean metric and, as such, maintains nonlinear features of the original data that are lost in traditional empirical orthogonal function analysis [44]. The geodesic distance employed by Isomap can be described by Swiss Roll, which is shown as Figure 12. In Figure 12a, the blue dotted line represents the Euclidean distance between two sample points, however, it cannot reflect the “real distance” of the two samples. The geodesic distance expressed by the blue line can truly reflect the distance of the two samples which are in the manifold space. The red line in Figure 12b is the approximate geodesic distance of the two samples which is calculated by the shortest path algorithm. We can see in Figure 12c, in two-dimensional spatial distribution, the approximate geodesic distance expressed by red line is really close to the real distance expressed by the blue line.

In Isomap, the followed method is utilized to approximately calculate the real geodesic distance. For a sample point in the data set, the geodesic distance in its neighborhood is replaced by Euclidean distance, while the geodesic distance out of its neighborhood is replaced by the shortest path on manifold. The shortest path can be calculated by Dijkstra algorithm or Folyd algorithm. The main steps of Isomap are as follows:

1. Structure neighborhood relation graph G(V,E)

For each sample point $x_{i}\left(i=1,2,\cdots ,N\right)$, the Euclidean distance between the sample and the other sample points is calculated. When $x_{j}$ is one of the k-nearest points to $x_{i}$ or the Euclidean distance $d\left(x_{i},x_{j}\right)$ between $x_{j}$ and $x_{i}$ is less than a fixed threshold ε, $x_{i}x_{j}$ is regarded as the edge of graph G, and the weight of edge $x_{i}x_{j}$ is $d\left(x_{i}x_{j}\right)$.

2. Calculate the shortest path

Supposing the shortest path is $d_{G}\left(x_{i},x_{j}\right)=d\left(x_{i}x_{j}\right)$ when G has an edge $x_{i}x_{j}$, otherwise $d_{G}\left(x_{i},x_{j}\right)=\infty$.

(17) $$d_{G}\left(x_{i},x_{j}\right)=\min\left\{d_{G}\left(x_{i},x_{j}\right),d_{G}\left(x_{i},x_{l}\right)+d_{G}\left(x_{l},x_{j}\right)\right\}$$

then the shortest path distance matrix $D_{G}={\left[d_{G}^{2}\left(x_{i},x_{j}\right)\right]}_{i,j=l}^{N}$ is obtained.

3. Calculate embedding dimensionality

The last step applies the classical MDS to the distance matrix $D_{G}$, constructing the d-dimensional embedding space Y while retaining the expected intrinsic geometric features of the manifold. This is achieved by minimizing the cost function:

(18) $$E={\left\|\tau \left(D_{G}\right)-\tau \left(D_{Y}\right)\right\|}_{L^{2}}$$

where matrix transformation operator $\tau \left(D_{G}\right)=-\frac{HSH}{2}$, and S is the matrix that contains the square of each element in D. $H=I-ee^{T}/n$, $e={\left[1,1,\cdots ,1\right]}^{T}$. Denote the eigenvalue of $\tau \left(D_{G}\right)$ as $s_{1},s_{2},\cdots$(descending order), and $v_{1},v_{2},\cdots$ is the corresponding column vector. Thus, the row vector of $Y=\left[\sqrt{s_{1}v_{1}},\sqrt{s_{2}v_{2}},\cdots ,\sqrt{s_{d}v_{d}}\right]$ is the most suitable d-dimensional embedded coordinate.

### 2.4. Insprition from Visual Cognition

Based on the above introduction of related theories, we can know that the recurrence plot has good performance in processing non-station and non-linear vibration signals. Therefore, a two-dimensional image can be transformed from vibration signals based on recurrence plot technique, which can reflect the fluctuation of working conditions on the variation of image scale, illumination, translation, and so on.

As a representative computing method based on VIC of the HVS, SURF can successfully find the stable points in images, which have strong robust to image scale, illumination, translation, etc. Inspired by this, stable fault features contained in the transformed two-dimensional images can be extracted by SURF method, which are also robust to variable working conditions.

Considering the SURF fault feature vector have a high dimensionality, which may result in high computation consumption, the manifold learning method, Isomap, can be utilized to excavated low-dimensional manifold structure hidden in the high-dimensional dataset. Finally, the stable fault feature vector is formed.

Based on the extracted low-dimensional stable fault feature vector, a fault classifier can easily realize fault diagnosis for rolling bearing under variable working conditions.

## 3. Method for Rolling Bearing Fault Diagnosis under Variable Conditions Based on Visual Cognition

**Figure 13:**
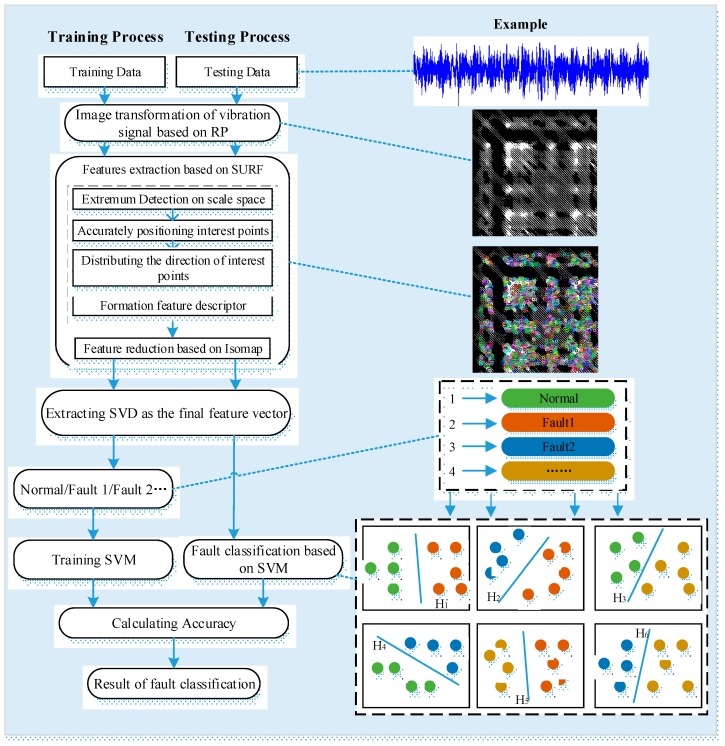
Flow diagram of the proposed method.

We utilize the following four steps, which are shown in Figure 13, to execute fault diagnosis under variable conditions. First, all of the vibration data under different conditions are transformed into RPs, which facilitates the use of the visual cognitive method to extract features. Then, SURF extracts interest points of all RPs and generates descriptors for fault diagnosis. Nevertheless, because of the fault intrinsic features embedded in the high-dimensional space, an intrinsic manifold is established using Isomap. The features of different fault modes under the same condition are used to train the SVM classifier and classify the fault mode under the other conditions, after which cross validation is indispensable.

### 3.1. Description of the Rolling Bearing Experimental Data

**Figure 14:**
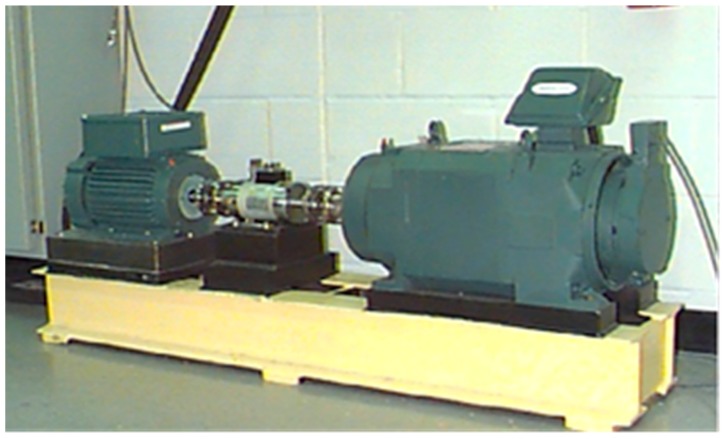
Test-rig of the rolling bearing.

Rolling bearing data collected by the Case Western Reserve University Bearing Data Center are used for testing and verifying the proposed method in the experiment [45]. As shown in Figure 14, the test-rig consists of a 2 hp (1 hp = 735 W) motor (left), a torque transducer/encoder (center), a dynamometer (right), and control electronics (not shown) [46]. The test-rig also includes both the drive end (DE) and fan end (FE) bearings of the 6205-2RS JEM SKF and NTN equivalent bearings, on which the single point faults were introduced using electro-discharge machining with fault diameters of 7 mils, 14 mils, 21 mils, 28 mils, and 40 mils (1 mil = 0.001 inches, 1 inch = 0.0254 m), respectively. To quantify the outer raceway fault stationary effect, experiments were conducted for both the FE and DE bearings with outer raceway faults located at 3 o’clock, 6 o’clock and 12 o’clock.

For data acquisition, vibration data under the normal, inner-race fault, outer-race fault, and rolling element fault conditions were collected by means of accelerometers, which were attached to the housing with magnetic bases and placed at the 12 o’clock position at both the drive end and fan end of the motor housing. Digital data, which were post-processed in a Matlab environment, were collected at 12,000 samples and 48,000 samples per second for the DE and FE bearing faults, respectively [46].

In this test-rig, the motor speed was varied between 4 conditions. To verify the efficiency of the proposed approach, the typical data were selected (4.8 kHz sampling rate, 0.021 inch and outer race fault at 6 o’clock) and are described in Table 1. The speed was varied to change the conditions of the bearings so as to realize data collection under variable conditions.

### 3.2. Image Transformation of Vibration Signals for Visual Cognition

The vibration signals of the rolling bearings are one of the easier acquired signals contained in the masses of information of rolling bearings. Choosing an appropriate signal processing method can allow for obtaining the required features and contribute to the fault diagnosis. To explore the features of different failure modes under variable conditions using these vibration signals, the vibration data were transformed into an image for visual cognition. As previously mentioned, RPs can uncover hidden periodicities in a signal in the recurrence domain, which are not easily noticeable, and it is important that the method analyzes the periodic, chaotic and non-stationary elements of the time series; thus, RPs are very suitable for the image transformation of vibration signals without loss of signal information.

Because of the need for calculating the ergodic Euclidean distance of $x_{i}$ and $x_{j}$ in the reconstructed phase space and the restriction of the computer calculation speed, only 1000 points of vibration signals are chosen each time to transform into an RP, and the transformed RP is a black-white image with dimensionalities of N×N (the sizes of different fault modes under different conditions show very slight differences).

### 3.3. Feature Extraction Based on SURF and Isomap

This section describes the feature extraction method of vibration data under variable conditions based on SURF. By utilizing SURF, the transformed images from the vibration signals are extracted interest points, which are described with directions and vectors. The direction is determined by the longest vector, which is the sum of all points of the Haar wavelet responses in the x and y directions within the neighborhood around the interest point within a sliding orientation window of 60°. The descriptor is described in the 4×4 sub-regions with a vector $v=\left(\sum d_{x},\sum \left|d_{x}\right|,\sum d_{y},\sum \left|d_{y}\right|\right)$, where $\sum d_{x}$ and $\sum d_{y}$ are the sums of the Haar wavelet response in the x and y directions in each sub-region, and $\sum \left|d_{x}\right|$ and $\sum \left|d_{y}\right|$ are the sums of the absolute values of the Haar wavelet responses in the x and y directions in each sub-region, respectively. Therefore, the descriptor is a 64-dimensional vector.

Due to the visual information appearance on the intrinsic manifold, which is embedded in the high-dimensional space $R^{m}$ constructed by the high-dimensional descriptor, the aforementioned Isomap method is employed to reduce the number of dimensionalities. Given an arbitrary point in $R^{m}$, the corresponding data point representing the fault mode on the intrinsic manifold can be obtained through mapping $g=f^{-1}$. However, the features of the low-dimensional intrinsic manifold are also too large and complex to be taken as feature vectors. To solve this problem and improve the robustness of the feature vectors, singular value decomposition (SVD) was utilized in this paper to compress the scale of the fault feature vectors and obtain more stable feature vectors. Therefore, mapping $g=f^{-1}$ from the high-dimensional space to the intrinsic manifold could give an SVD scatter diagram that can be shown in three-dimensional space by selecting the first three dimensionalities.

### 3.4. Fault Classification Based on SVM

In this paper, the classic classifier SVM is selected to classify the extracted features from vibration signals, which are processed by SURF. The libsvm toolbox in Matlab, which was developed by Zhiren Lin, was used to find the optimal classification plane H of different fault modes. In terms of multi classification, the libsvm toolbox searches for the hyperplane between two arbitrary types of fault features through the one-versus-one method, and then the classifier is established. Next, the features of four types of fault modes under the same condition are employed as training data to classify the features of the other three conditions, and cross validation is also necessary.

## 4. Results and Discussion

To verify the fault diagnosis under variable conditions, the vibration data of the 4.8 kHz sampling rate and 0.021 inch under four different speeds are utilized. The normal, inner race fault, outer race fault and rolling element fault vibration data under four different conditions are equivalently represented by RPs and 20 sets of vibration data which contains 1000 points are selected for each fault mode under each condition. For each set of vibration data, Cao algorithm and mutual Information Method are employed to select the appropriate embedded dimensionality m and delay time τ to reconstruct the phase space of the time series of vibration signals. The parameters m and τ in each conditions are shown in Table 2. To analyze the influence of conditions on RPs, for each fault mode of four different conditions, a set of test data are randomly selected to generate RPs for comparison and analysis.

**Figure 15:**
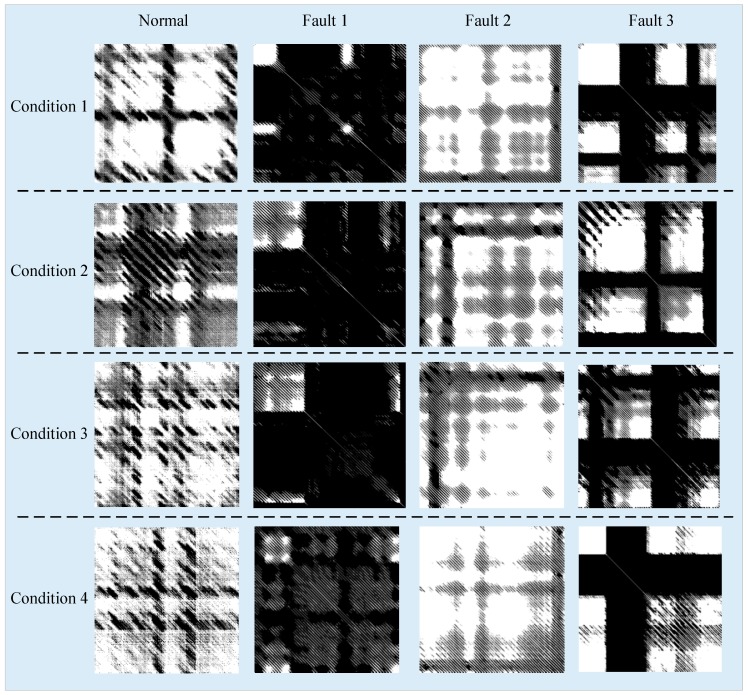
RPs transformed by fault mode vibration data under different conditions.

The transformed RPs are shown in Figure 15. Different rows represent different conditions; the first column shows the RPs in the normal state, the second column shows the RPs in the inner fault state, the third column shows the RPs in the rolling element fault state, and the fourth column shows the RPs in the outer fault state.

In Figure 14, we can see that the RPs of different fault mode under different conditions have different structural characteristics, while the same fault mode under different conditions have a strong similarity structure. Affected by the change of conditions, RPs under different conditions show the translation variation (shown as the first column of the third row and the fourth row), scale variation (shown as the second column of the first row and the second row) and the combination of these changes.

**Figure 16:**
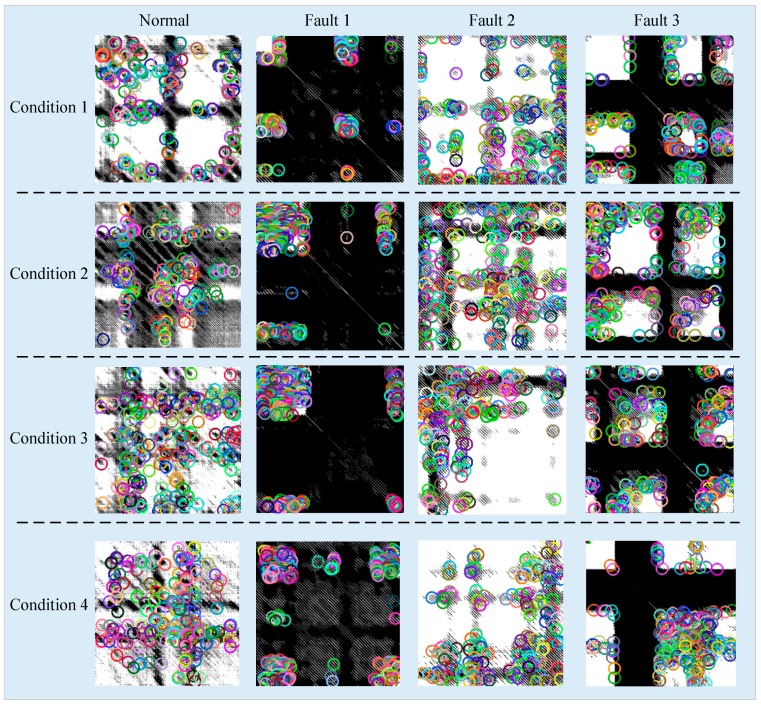
Detected interest points in RPs.

After the image transformation, SURF is employed to extract features from the transformed images. The extracted interest points under variable working conditions of each failure mode are shown in Figure 16.

Each RP generates a 64-dimensional SURF feature vector after the feature extraction. Since the dimensionality of the feature vector is very high, it may cause huge computational consumption and low diagnosis accuracy. According to MPC, the low-dimensional manifold space can effectively highlight the features which are not easy to explore in high dimensional space, therefore Isomap is employed to reduce the feature dimensionality. To explore the relationship between the classification accuracy and the reduced dimensionality, the 64-dimensional feature vector is reduced to two-dimensional (2-D), 3-D, 5-D, 8-D, 10-D, 12-D, 15-D, 18-D, and 20-D, respectively.

SVM is employed as the fault classifier to realize the fault classification of the rolling bearing under different working conditions. Cross validation is utilized to verify the accuracy of the proposed method; that is, the vibration data collected under each condition are orderly selected as training data, and the data collected under the other three conditions are as test data, as shown in Table 3.

In each cross validation, the training data and test data are composed as follows:

Training data: 20 groups of data of each fault mode under one condition, totally 80 groups of data.

Test data: 20 groups of data of each fault mode under the other 3 conditions, totally 240 groups of data, where 1–80 groups of data are the first condition, 81–160 groups of data are in the second condition, and 161–240 groups of data are in the third condition.

**Figure 17:**
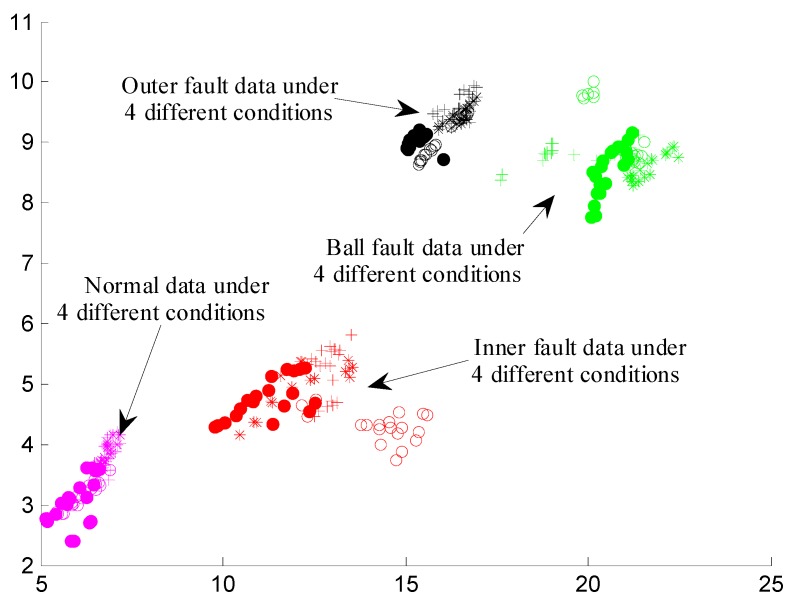
The feature scatter diagram in two-dimensional space.

**Figure 18:**
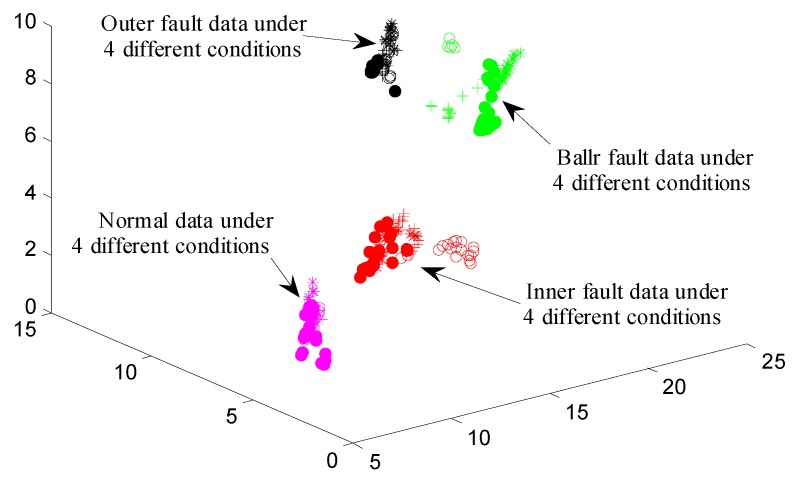
The feature scatter diagram in three-dimensional space.

Table 4 shows the classification accuracy of the cross validation in different dimensionality. From Table 4, we can see that the lowest classification accuracy value is 92.083%. Moreover, the classification accuracy increases with the reduced dimensionality increasing. The classification accuracy reaches the highest when the reduced dimensionality reaches 8, in which the classification accuracy values of four groups of cross validation are above 99%, indicating that the correct fault detection probability is very high. To visually display the features of different fault modes under different conditions, the authors draw the feature scatter diagram in the two-dimensional and three-dimensional space, as shown in Figure 17 and Figure 18, respectively.

## 5. Conclusions

This study proposes a fault diagnosis method for rolling bearings under variable conditions based on visual cognition. First, this method transforms the vibration signals into images, which are expressed in RPs. The core step is to employ the VIC of the HVS to extract the stable features of different RPs transformed from vibration signals under different working conditions. After that, we utilize the Isomap method to establish the intrinsic manifolds embedded in the high-dimensional space, which reveal the clusters of features. Lastly, the classical classification method SVM is employed to execute fault diagnosis.

The proposed fault diagnosis method is a multidisciplinary method which successful introduces visual cognition into the field of fault diagnosis. From the experiment results based on data from the bearing data center of Case Western Reserve University, the following conclusions can be reached:

(1) Inspired by VIC of the HVS, stable fault features contained in the vibration signals collected under variable working conditions can be successfully extracted by SURF algorithm.
(2) Inspired by MPC of the HVS, the manifold learning method, Isomap, can successfully excavated meaningful low-dimensional structures hidden within high-dimensional observations.

This study provides a promising approach to machine fault diagnosis from the cognitive computing field, especially for those rotating machineries which usually work in a variable working condition. Therefore, it is of great significance to engineering practice.

In the future, more characteristics of the HVS and the possibility of being introduced into the field of fault diagnosis could be investigated. Moreover, the authors plan to apply this method to more types of rotating machineries to further verify its general applicability.

## Figures and Tables

**Table 1:** Experiment parameters of each fault mode under different working conditions.

Fault Diameter	Motor Speed	Inner Race	Rolling Element	Outer Race		
(inch)	(rpm)			3 o’clock	6 o’clock	12 o’clock
0.021	1797	213.mat	226.mat		238.mat	
	1772	214.mat	227.mat		239.mat	
	1750	215.mat	228.mat		240.mat	
	1730	217.mat	229.mat		241.mat	

**Table 2:** The experiment parameters of each fault mode under different conditions.

Conditions	Parameters	Normal	Inner Race Fault	Rolling Element Fault	Outer Race Fault
Condition 1	m	15	12	13	12
	τ	4	5	5	5
Condition 2	m	15	12	12	12
	τ	4	5	5	5
Condition 3	m	15	20	13	12
	τ	4	2	5	5
Condition 4	m	16	11	13	12
	τ	4	5	5	5

**Table 3:** The data composition of rolling bearing under variable conditions for cross validation.

Groups of Cross Validation	Conditions of Training Data	Conditions of Test Data		
1	1	2	3	4
2	2	1	3	4
3	3	1	2	4
4	4	1	2	3

Note: 1, 2, 3, 4 in the conditions of training data and test data denote four different speed conditions, 1797 rpm, 1772 rpm, 1750 rpm and 1730 rpm, respectively.

**Table 4:** Classification accuracy of the cross validation in different dimensionality.

Groups of Cross Validation	Classification Accuracy								
	2-D	3-D	5-D	8-D	10-D	12-D	15-D	18-D	20-D
1	99.166	99.166	100	100	100	100	100	100	100
2	99.166	99.166	99.166	100	100	100	100	99.583	99.166
3	92.083	94.588	97.083	99.166	99.166	99.166	99.166	99.166	99.166
4	92.5	95.416	100	99.583	99.583	99.583	99.583	99.583	99.583
